# Environmental impact of anesthetic gases

**DOI:** 10.4103/mgr.MEDGASRES-D-25-00243

**Published:** 2026-01-23

**Authors:** Lesley Bennici, Hana Mucevic, Jing Tong, Ana Costa

**Affiliations:** 1 Department of Anesthesiology, Renaissance School of Medicine at Stony Brook University, Stony Brook, NY, USA; 2 Renaissance School of Medicine at Stony Brook University, Stony Brook, NY, USA

**Keywords:** anesthetic gases, environmental impact, global warming, greenhouse effect, greenhouse gases, low flow anesthesia, minimum alveolar concentration, sustainability, total intravenous anesthesia, volatile anesthetics

## Abstract

**Facts**

1. Healthcare systems negatively impact the environment, and the operating room is a major contributor.
2. Inhalational anesthetics contribute to hospital greenhouse gas emissions.
3. Understanding the pharmacology and clinical applications of commonly used anesthetic gases allows for meaningful discussion of environmental impact mitigation strategies, balanced with quality patient care.

**Open Questions**

1. What are the commonly accepted definitions of low flow anesthesia and how can they be implemented clinically?
2. How can we best quantify the environmental impact of anesthetic gases and hypnotic agents in total intravenous anesthesia
3. What are available strategies to mitigate the detrimental effects of anesthetic gases to the environment?

Healthcare systems are known to negatively impact the environment, with the operating room significantly contributing to these issues. Specifically, anesthetic gases have been a recent target of many sustainability initiatives, as they are known to be greenhouse gases and have traditionally been a cornerstone of providing general anesthesia. This review focuses on the current literature regarding the impact of anesthetic gases on the environment, including common definitions such as global warming potential and carbon dioxide equivalents. The most commonly used anesthetic gases are reviewed, including their impact on the atmosphere, as well as strategies to reduce their negative impact while maintaining their availability for use in the practice of anesthesiology. Specifically, the clinical applications of the commonly used anesthetic gases, namely sevoflurane, desflurane, isoflurane and nitrous oxide are discussed. This review further identifies and explores alternative methods that help mitigate the negative environmental impact of anesthetic gases, including gas capturing systems, low flow anesthesia and total intravenous anesthesia, as well as barriers to implementing these strategies. We conclude that while many strategies exist to minimize the environmental impact of anesthetic gases, implementation is often hindered by factors such as institutional buy-in and cost/return on investment ratio.

## Introduction

Healthcare systems are known to negatively impact the environment.^1^,^2^,^3^,^4^ Activities such as heating, ventilation, and air conditioning, pharmaceutical and medical equipment usage, transportation, laundry, and electronic use require finite environmental resources, emit pollutants, and generate waste.^1^ The healthcare sector has continued to contribute to the world’s greenhouse gas emissions with an increase by 36% since 2016.^5^ In the United States, healthcare produces an average of 8.5% of total greenhouse gas emissions, contributing substantially to climate change.^2^ Approximately 3% of healthcare’s greenhouse emissions are attributed to volatile anesthetics in high-income nations.^6^ These pressures on the environment can increase human contact with toxins in the air, soil, or water, and in turn negatively impact human health.^1^,^2^ The disturbances seen in climate change increase the likelihood of heatwaves, floods, and droughts, which also elevate the risk of disease and need for trauma care.^4^,^7^ As countries with lower human development index scores tend to have higher proportions of outdoor workers, the labor forces in these underserved regions are disproportionately impacted by these global trends.^5^

Surgery and anesthesia are uniquely resource-intensive health services due to the heavy use of medical devices as well as marked energy and water demands.^4^,^8^,^9^ Accordingly, the operating room significantly contributes to the environmental impact of healthcare.^4^ Inhalational anesthetics are anesthetic gases that are used intraoperatively to provide general anesthesia and are estimated to make up 5% of hospital greenhouse gas emissions.^9^,^10^,^11^ Traditionally, they have been a cornerstone of general anesthesia as they are easy to use, and their effects are well-studied and predictable. In modern practice, anesthetic gases are commonly used in all age groups for a multitude of surgeries and procedures, as well as in situations where intravenous access is challenging overall.^11^ However, inhalational anesthetics are potent greenhouse gases.^9^ Desflurane, for example, has over 2500-times the heat-trapping ability of carbon dioxide (CO_2_).^11^ Furthermore, some anesthetic gases like nitrous oxide (N_2_O) and isoflurane can deplete ozone in the atmosphere, reducing the Earth’s atmospheric protection from ultraviolet radiation.^12^ Although anesthetic gases are metabolized at different rates, primarily by the cytochrome p450 system, modern volatile anesthetics are metabolized to a minimal extent.^13^ 95% of inhaled anesthetics are exhaled unchanged, scavenged in the operating room to prevent occupational exposure, then later released into the atmosphere.^11^,^12^ These gases can stay in the atmosphere for years, extending their impact on global warming.^11^,^14^ Not only is N_2_O the single greatest contributor to ozone depletion, it can also persist in the atmosphere for over a century.^11^

**Figure 1:**
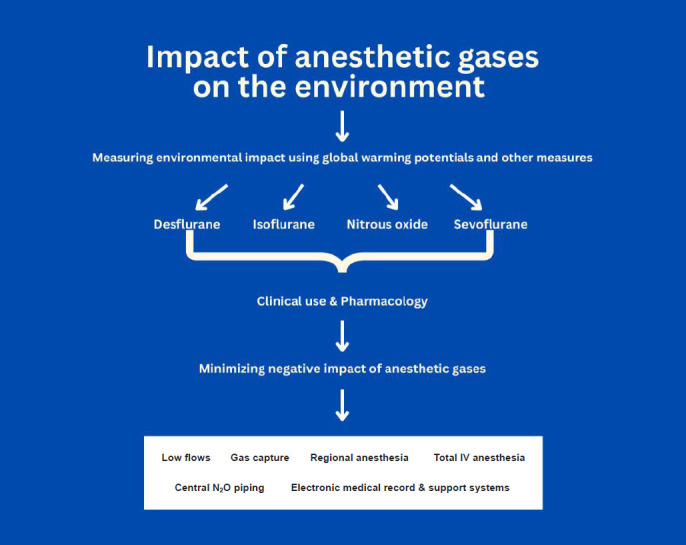
Design of environmental impact on anesthetic gases. ©Jing Tong via Canva.com. N_2_O: Nitrous oxide; IV: intravenous.

In light of considerable environmental repercussions and sustainability initiatives in healthcare, anesthetic regimens can be modified to decrease their environmental impact without affecting the quality of patient care.^4^,^12^,^14^ New methods have been proposed, such as applying low flow rates, specifically minimal or metabolic flow rates, improving gas management, such as efficient scavenging systems and ensuring well-maintained equipment to prevent leaks, and limiting the use of gases with high global warming potential (GWP).^9^,^11^ Alternatively, intravenous or regional anesthesia may be used in place of inhalational agents when appropriate.^4^,^14^ This review explores the environmental impact of common anesthetic gases and highlights a few measures that help mitigate their detrimental impact. More importantly, it considers the detrimental impact of anesthetic gases to the environment and proposes ways to mitigate this negative impact while also providing specific clinical considerations in anesthetic practice that do not compromise high-quality patient care (**Figure 1**).

## Methods

This review was conducted using a narrative review methodology. A comprehensive literature search was performed across multiple databases, including PubMed, Scopus, Google Scholar and Web of Science to identify peer-reviewed articles related to the environmental impact of anesthetic gases. Keywords used in the search included “anesthetic gases,” “volatile anesthetics,” “environmental impact,” “greenhouse gases,” “sustainability in anesthesia,” “global warming potential,” “desflurane,” “sevoflurane,” “isoflurane,” “total intravenous anesthesia (TIVA),” “nitrous oxide,” “low flow anesthesia,” “anesthetic gas scavenging,” “gas capture systems,” “electronic medical record,” “cost” and “economic.” Boolean operators (AND/OR) were applied to refine search results. The search was limited to articles published in English from 2000 to 2025. Additional references were identified through manual searches of the bibliographies of relevant articles. Inclusion criteria encompassed original research articles, systematic reviews, meta-analyses, and policy statements that addressed the environmental effects of anesthetic gases or strategies to mitigate their impact. Exclusion criteria included editorials, and studies not directly related to anesthetic gas emissions or sustainability. Data from the included studies were synthesized narratively, with emphasis on comparing the GWP, atmospheric lifetime, economic feasibility and clinical utility of various anesthetic agents. Strategies for reducing environmental impact, such as low-flow anesthesia, gas capture technologies, and the use of total intravenous or regional anesthesia, were also reviewed and summarized based on the available evidence.

## Environmental Impact Measure of Global Warming

Global warming refers to a sustained increase in the Earth’s average surface temperature.^12^ As sunlight enters the Earth’s atmosphere, greenhouse gases absorb its radiation. In this way, greenhouse gases such as CO_2_ trap heat emitted by the sun and raise the Earth’s surface temperature.^12^ The potential of a gas to trap heat in the atmosphere is quantified by its GWP, which measures how much energy 1 ton of a particular gas will absorb over a given period of time, usually 100 years (GWP_100_).^4^,^12^ GWP is measured in CO_2_ equivalents (CO_2_e) as CO_2_ comprises 86% of greenhouse gas emissions.^7^ CO_2_ is utilized as a reference compound in these calculations, and halogenated anesthetics decay more rapidly in the atmosphere than CO_2_ and have GWPs that decrease markedly with increasing time horizons.^15^ A carbon footprint broadly describes the estimation of the indirect and direct greenhouse gas emissions associated with a process, product, or organization, which is also quantified relative to CO_2_ in CO_2_e.^16^ The specific calculation of carbon footprints can vary; for example, to measure the carbon footprint of a particular drug, a life cycle assessment may be performed to measure the total sum of greenhouse gas emissions associated with the production, use, and disposal of each container of that drug.^17^

## Clinical Properties and Applications of Inhaled Anesthetics

**Figure 2:**
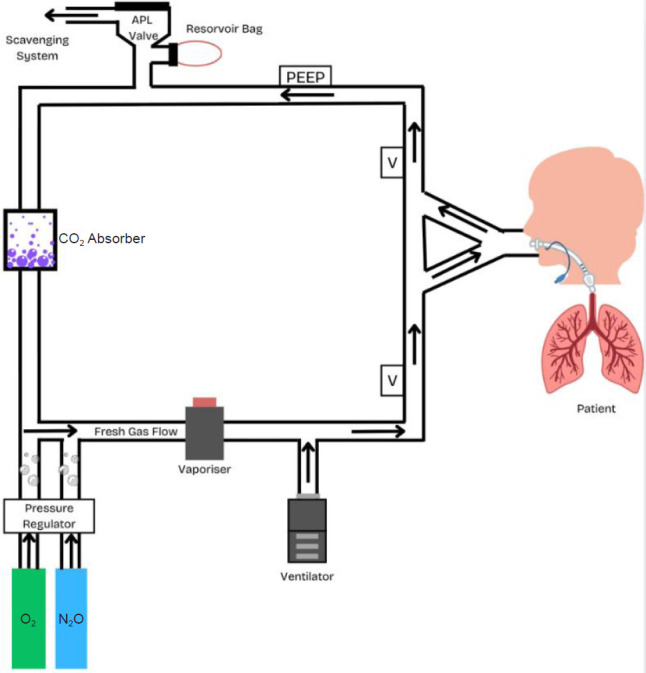
A diagram of the anesthesia machine. ©Hana Mucevic via Canva.com. APL: Adjustable pressure limiting; CO_2_: carbon dioxide; N_2_O: nitrous oxide; O_2_: oxygen; PEEP: positive end-expiratory pressure; V: ventilation.

Surgeries requiring general anesthesia have become extremely common, with general anesthesia being administered to more than 300 million patients worldwide each year.^18^ The American Society of Anesthesiologists defines general anesthesia as “a drug-induced loss of consciousness during which patients are not arousable, even by painful stimulation.”^19^ General anesthesia can be achieved by utilizing pharmacologic agents, in the form of intravenous and/or inhaled medications. Volatile inhaled anesthetic gases have traditionally been a cornerstone of general anesthesia, with the more recent emergence of TIVA, often using the medication propofol, as an alternative. The most common anesthetic gases utilized across the globe are N_2_O and the highly fluorinated gases sevoflurane [(CF_3_)_2_CHOCH_2_F], desflurane (CF_3_CHFOCHF_2_) and isoflurane (CF_3_CHClOCHF_2_). Typically, these gases are inhaled by patients via a mask or airway device and then exhaled with their chemical structures largely unchanged by the patient’s metabolism. The exhaled excess gas is directed to a waste scavenging system, to which the anesthesia machine is connected, and then redirected into the atmosphere (**Figure 2**).

While any of these anesthetic gases can be used when maintaining a state of general anesthesia, clinically they each have certain properties that make them more useful in specific scenarios. When discussing the properties of anesthetic gases, it is important to understand the concept of minimum alveolar concentration (MAC), which is unique to each anesthetic gas and reflects the lowest concentration of an inhaled anesthetic that is required to prevent movement in 50% of patients when exposed to a painful surgical stimulus (a MAC of 100%).^20^ MAC is inversely related to potency, with anesthetic gases that have a higher MAC being less potent (a higher concentration of gas is required to achieve the same clinical effect).^20^ To achieve a MAC of 100%, N_2_O would require a concentration of 104%, thus making it less potent and unable to be used as a sole anesthetic. It is, however, a potent adjunct due to its analgesic and anesthetic properties. It also has low blood solubility, which provides it with quick onset and offset actions. It has minimal effects on respiratory drive and hemodynamic stability, and it also serves to further concentrate other volatile anesthetics in the lungs, an effect known as the “second gas effect.”^21^ For all of these reasons, N_2_O is widely used globally, as an adjunct for general anesthesia, for procedural sedation as a sole agent (often in dental clinics), and on labor and delivery floors for analgesia.

Sevoflurane has become a popular choice for the induction and maintenance of general anesthesia. With a MAC value of around 2.1% (varies based on patient age), it is fairly potent and can be used as a sole general anesthetic agent. Its sweet smell and low pungency make it useful in the setting of inhalational inductions, particularly in the pediatric population, where pre-induction intravenous access may be an issue.^22^ One of the potential issues that is often discussed pertaining to sevoflurane is the formation of the nephrotoxic compound A. In animal studies, dose and length of exposure related effects of sevoflurane were noted to cause the formation of compound A, which caused mild and reversible renal injury. This has never been demonstrated in humans but has often been the reason that clinicians choose to use moderate or high fresh gas flows (FGF) when using sevoflurane. Notably, when higher FGFs are used during maintenance of general anesthesia with volatile anesthetics, more of the anesthetic gas is utilized and collected as waste for disposal. Sevoflurane has not been shown to cause elevations in creatinine and blood urea nitrogen, markers of renal function, above the upper limit of normal in healthy adults without coexisting renal disease.^23^

Desflurane, due to its low blood solubility, has a quicker onset and offset of action when compared to sevoflurane. It does not result in compound A formation, and can be run at low FGF, leading to a smaller waste footprint. However, it is extremely pungent and contraindicated for use in inhalational inductions due to the risk of airway irritability. It has a MAC value of 6.0% (varies with age), making it less potent than sevoflurane, but it too can be used as a sole agent for maintenance of general anesthesia. Of note, the vapor pressure of desflurane is 681 mmHg at sea level, which is significantly higher than that of other volatile anesthetics. This leads to a boiling point near room temperature, necessitating the use of a special vaporizer to deliver the medication. The vaporizer requires minimal electricity, even though the anesthetic is kept heated at all times.^24^ Desflurane can also cause a clinically significant, albeit transient, increase in heart rate secondary to catecholamine release, which may be detrimental in certain patient populations.^25^ In addition to greenhouse emissions, fluorinated volatile anesthetics such as sevoflurane and desflurane are classified as per- and polyfluoroalkyl substances, contributing long-term environmental pollution in aquatic systems via their degradation products, namely hexafluorisopropanol and trifluoroacetic acid.^26^

Isoflurane, approved for use in the United States in 1979, has been in use longer than both sevoflurane and desflurane. Like its predecessor, enflurane, isoflurane is non-flammable, but it is pungent and contraindicated for use in inhalational induction. It has a MAC of 1.2% (varies with age), making it slightly more potent than sevoflurane and much more potent than desflurane. It can be used as a sole agent for maintenance of general anesthesia at a low FGF rate, leading to a reduced amount of waste. Of note, all of the inhaled anesthetics cause dose-dependent central nervous system depression, muscle relaxation, decreased blood pressure and cardiac output, reduced tidal volume and increased respiratory rate.^27^ The chemical structures of the common anesthetic gases are presented in **Table 1**.

**Table 1:** Chemical structure of the common anesthetic gases

Molecule	Structure
Desflurane	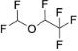
Isoflurane	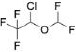
Sevoflurane	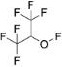
Nitrous Oxide	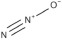

## Environmental Impact of Nitrous Oxide and Volatile Anesthetic Gases

In recent years, these gases have been identified as greenhouse gases and discovered to contribute negatively to climate change. N_2_O and the halogenated gases that contain chlorine or bromine, such as isoflurane, have been found to deplete the ozone layer, thus diminishing its ultraviolet radiation-shielding effects.^28^ The other highly fluorinated gases that do not contain chlorine or bromine, namely sevoflurane and desflurane, have been found to absorb and reduce outgoing infrared thermal energy, thus trapping heat and warming the environment.^28^ GWP depends upon two factors: radiative efficiency and the atmospheric lifetime of the gas.^15^ Radiative efficiency depends on the strength and position of the gas’s infrared absorption bands and reflects the change in solar energy irradiance on the Earth’s atmosphere that occurs with a change in concentration of the compound in question.^29^ The halogenated anesthetic gases in question are similar in terms of radiative efficiency, with sevoflurane performing the best at about 25% less effect on solar energy irradiance than isoflurane and desflurane.^29^ Atmospheric lifetime depends upon how rapidly a compound is broken down, particularly by the hydroxy (OH-) radicals in the atmosphere. The bond between carbon and fluoride (C-F) is stronger than the bonds between carbon and hydrogen (C-H), chlorine (C-Cl) or bromine (C-Br). Thus, hydroxy radicals in the atmosphere are less able to displace the fluorine atoms bonded to carbon in the halogenated anesthetic gases. Furthermore, hydroxy radicals are also able to react more easily with halocarbon molecules, where carbon atoms are bonded directly to other carbon atoms. The reactivity of halocarbon molecules to hydroxy radicals is directly related to the number of carbon atoms bonded to the same carbon atom; that is, a carbon atom bonded to two carbon atoms reacts more with a hydroxy radical than a carbon atom bonded to a single carbon atom. Thus, sevoflurane has an atmospheric lifetime of 1–2 years, isoflurane of 3 years, and desflurane of 14 years.^29^ To put these GWPs in context, one hour of anesthesia with desflurane is equivalent to automobile emissions from driving a distance of several hundred miles, whereas 1 hour of isoflurane or sevoflurane anesthesia is equivalent to around 30 miles and 18 miles, respectively (FGF rates 0.5–2.0 L/min).^28^

Nonetheless, inhalational anesthetics make up only a small portion of total greenhouse gas emissions. In 2012, the total annual greenhouse gas emissions in the United States were 6.2 gigatons of CO_2_e, of which 6.8% was made up of N_2_O and fluorinated volatile anesthetic gases.^28^ Between 2011 and 2013, the contributions of N_2_O and inhalational anesthetics to greenhouse gas emissions from the healthcare sector were about 1%, representing only 0.1% of total United States greenhouse gas emissions.^28^ Given the large number of general anesthetics performed globally each year, it remains important to evaluate ways to minimize or negate the impact of volatile inhalational anesthetic gases, both to minimize occupational exposure as well as improve environmental health.

## Improving the Environmental Impact of Inhaled Anesthetics

### Environmental considerations

**Figure 3:**
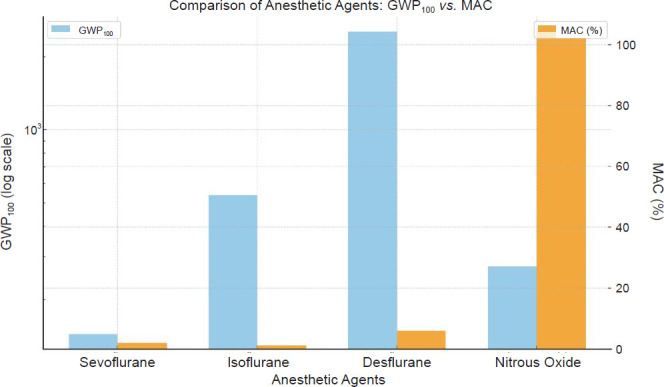
Comparison GWP100 and MAC of commonly used anesthetic gases. GWP_100_: Global warming potential over 100 years; MAC: minimum alveolar concentration.

Current research has indicated that desflurane has the greatest environmental impact, due to its significant contribution to greenhouse gas emissions, when compared to other inhaled anesthetics.^29^,^30^,^31^ Desflurane is believed to make up 80% of the combined greenhouse effect of all volatile anesthetics.^30^ Over a 100-year period, desflurane has a GWP that is approximately 40–50 times greater than that of isoflurane and sevoflurane.^31^ The main indicator of environmental impact caused by desflurane, sevoflurane, isoflurane, and N_2_O is GWP.^29^,^30^,^31^ The most recent values of GWP_100_ amongst the inhaled anesthetics are 2.540 for desflurane, 539 for isoflurane, 273 for N_2_O and 144 for sevoflurane.^29^,^30^,^31^ Based on GWP_100_, sevoflurane is the best inhaled anesthetic gas to use if the desire is to limit environmental impact caused by direct greenhouse gas emissions.^29^,^30^,^31^ The cradle-to-grave life cycle of each inhaled anesthetic offers another lens through which to assess the overall environmental impact of each gas.^30^,^32^ Aside from direct gas emissions, inhaled anesthetics can contribute to global warming through resource extraction, drug manufacturing and transportation, clinical use and drug disposal. Even when accounting for the cradle-to-grave life cycle, desflurane produced the highest greenhouse gas emissions.^32^ When based on the functional unit equivalent of one MAC of the gas required per hour, emissions of desflurane were 20 and 15 times greater than those of sevoflurane and isoflurane, respectively.^32^ An additional component of inhaled anesthetics that contributes to their environmental impact is their minimal *in vivo* metabolism. The lack of metabolism results in most of the inhaled anesthetics being exhaled by patients back into the atmosphere.^32^ Atmospheric lifetimes determine how long these gases stay in the atmosphere, contributing to global warming. The atmospheric lifetime of N_2_O is 114 years, much greater than that of other inhaled anesthetics. Desflurane follows with 14 years, then isoflurane with 3 years and sevoflurane with 1–2 years.^30^ Therefore, although the GWP_100_ for N_2_O is the second lowest, it spends a much larger time in the atmosphere, a part of the total greenhouse gases. The relationship of GWP_100_ and MAC for the common anesthetic gases is shown in **Figure 3**.

Several hospitals have already taken initiatives to eliminate the more environmentally potent greenhouse gases from the inhaled anesthetics they utilize. The Yale New Haven Health System and University of Wisconsin eliminated the use of desflurane.^31^ Yale’s largest hospital reduced their annual emissions in 2013 by 1600 tons CO_2_e. In 2015, the University of Wisconsin achieved a reduction of average emissions per anesthetic case from 163 kg CO_2_e to 58 kg CO_2_e.^31^ Hospitals continue to reduce their use of desflurane and N_2_O and replace them with environmentally favorable gases such as sevoflurane and isoflurane.

However, a recent study utilized mathematical models that made the case for the use of desflurane, arguing that metrics like GWP cannot be used to compare volatile anesthetics to CO_2_.^33^ The authors state that current desflurane emission rates will result in a very small, negligible increase of the global mean surface temperature increase of 0.00015°C, even if desflurane continues at the current usage for the next 100 years. In addition, the authors claim this small temperature increase is reversible in that the temperature increase will disappear in a few years if the use of desflurane is discontinued. The clinical and environmental properties of the common anesthetic gases are presented in **Table 2**.

**Table 2:** Clinical and environmental properties of common inhaled anesthetics

Inhaled anesthetics	MAC (approx, adults)	Potency	GWP_100_ (CO_2_ equivalents)	Atmospheric lifetime	Notes
Sevoflurane	2.1%	Moderate potency	144	1–2 yr	Least environmental impact; used for inhalation induction (esp. pediatrics); concern about compound A (no human toxicity proven).
Desflurane	6.0%	Least potent	2540	14 yr	Worst GWP among volatiles; quick onset/offset; needs special vaporizer; pungent (not for induction).
Isoflurane	1.2%	Most potent	539	3 yr	Low cost, pungent (not for induction); more environmentally harmful than sevoflurane but less than desflurane.
Nitrous oxide	104% (cannot achieve MAC 1 alone)	Weak; used as adjunct	273	114 yr	Major ozone-depleter; longest atmospheric persistence; large losses from central piping systems.

CO_2_: Carbon dioxide; GWP: global warming potential; GWP_100_: GWP over 100 years; MAC: minimum alveolar concentration.

### Low-flow anesthesia

The flow rates of inhalational gases contribute to their environmental impact. An FGF rate of 2 L/min has been common practice when using inhaled anesthetics, sevoflurane in particular, due to a theoretical risk of renal injury with lower flow rates.^31^ However, there has been no clinical evidence to support this theory in humans.^34^ In contrast, lower flow rates have been shown to have the potential of reducing the amount of inhaled anesthetic released into the atmosphere, effectively reducing its environmental contamination. Reducing isoflurane’s flow rate from 2 L/min to 1 L/min would prevent approximately 18,900 L of the gas from entering the atmosphere over a time span of 35 years if 500 cases were done per year.^35^ Reducing gas flow rates would require the increased use of CO_2_ absorbents, such as soda lime or calcium hydroxide-based compounds. Absorbents have a much lower environmental footprint, primarily contributing to solid waste.^35^ Studies highlight that while low-flow techniques increase absorbent use, they drastically reduce anesthetic gas emissions. This trade-off favors low-flow anesthesia as a more sustainable practice.^32^,^35^

While there is not universally accepted definition of low flow anesthesia, rates less than alveolar ventilation can be described as low flow.^36^ Baker^36^ has classified flow rates as: minimal 0.25–0.5 L/min, low 0.5–1 L/min, medium 1–2 L/min and metabolic approximately 0.25 L/min. Metabolic flow rates are generally accepted as rates that closely match the patient’s individual gas uptake and metabolic consumption, namely less than 0.35 L/min.^37^ Modern anesthesia machines have the capability to control for these rates with built-in alarm systems that alert the anesthesiologist when higher oxygen levels are needed as well as when target end-expiratory concentrations of the gas reach a threshold. It is recommended that 1 L/min or less, with a minimum of 0.25 L/min, be used in the maintenance phase of anesthesia.38 However, there are limited recommendations for low FGF rates that should be used during the induction and emergence phases of anesthesia.^35^ The induction phase of anesthesia tends to require higher FGF rates so that the desired concentration of the anesthetic can be reached in a short amount of time. Although higher FGF rates are indicated for induction, there is no benefit from high FGF rates once the desired inhaled agent’s concentration is reached. In general, FGF rates during induction must be greater than the patient’s minute ventilation, to prevent rebreathing of the gas, but only slightly.^35^ Any flow rates much greater than minute ventilation are unnecessary for induction and increase the amount of gas lost to the outside atmosphere. Additionally, most inductions of general anesthesia are currently accomplished with intravenous agents in the United States.^39^ Thus, inhalational inductions are typically reserved for patients who cannot tolerate intravenous access insertion prior to the anesthetic. Emergence, on the other hand, presents a challenging time as gas is lost to the atmosphere due to gas expulsion until recent gas-capturing system innovations.^35^

### Gas capturing technology

When using an inhaled anesthetic, the amount supplied typically exceeds the demand of the patient.^40^ To protect occupational health in operating rooms, scavenging systems are used to direct exhaled gases to the outside atmosphere, thus preventing these gases from being expelled into the operating room. Scavenging systems can be active, where the Venturi effect creates suction by accelerating a moving gas through a narrow constriction, thus utilizing negative pressure to remove waste gases from the system.^41^ Scavenging systems can also be passive, where the gas passively moves through tubing and into the ventilation system. Scavenging system interfaces are defined as open or closed, and most modern scavenging systems use open interfaces, which communicate with the outside environment.^39^ Due to the environmental consequences of releasing inhaled anesthetics to the outside world, gas recapturing technologies have entered the market. These systems directly adsorb the inhaled anesthetics or condense them into a liquid.^30^ CONTRAfluran™ Anesthetic Gas Capture System, produced by Baxter International (Deerfield, IL, USA), claims to capture 99% of the inhaled anesthetic when sevoflurane or desflurane are used.^42^ However, a study of the *in vivo* use of CONTRAfluran™ on 80 consecutive patients reported a desflurane capture rate of 25%, likely impacted by a low FGF rate of 0.8 L/min, which limited the volume of anesthetic available for capture.^42^ In a similar study, the volatile capture device was used on 43 patients, and there was a reported 43–51% capture rate when sevoflurane or isoflurane were used, though the absence of reported FGF rates raises concern about overestimation due to high-flow bias.^43^ Of note, a considerable amount of potent inhaled anesthetic is still stored in body tissues upon emergence and therefore is not available for capturing systems after extubation, leading to a capture rate lower than 99%. Blue-Zone Technologies, a Canadian company, offers two complementary solutions—Deltasorb® for individual operating rooms and Centralsorb® for centralized gas capture—that utilize zeolite-based canisters to adsorb WAGs.^44^ *In vitro* efficiencies of Blue-Zone’s systems can exceed 95%, but *in vivo* capture varies significantly, with studies reporting ranges from 25% to 73% depending on anesthetic agent, equipment configuration, and FGF rate.^45^ These captured agents can be reprocessed into generic anesthetic products, creating a closed-loop pharmaceutical supply model. Although gas-capturing technology has the potential to minimize the environmental contamination of inhaled anesthetics, there is minimal peer-reviewed evidence to support this.^30^,^43^ Additionally, capture rate may not be an adequate measure of efficiency as it is impacted by variables such as FGF rates, body composition and surgery duration.^43^ Furthermore, these capture systems have not had full life cycle assessments that evaluate the impact of manufacturing and transporting the device, as well as processing the waste anesthetic.^30^,^43^ Therefore, further studies are needed to fully assess the environmental benefit and economic feasibility of gas capture technology when compared to other efforts.^43^

In parallel with these environmental technologies, closed-loop anesthesia delivery systems represent a different, but complementary, strategy to reduce anesthetic waste and optimize patient care. These systems use real-time physiologic feedback, such as bispectral index, end-tidal anesthetic concentration, or hemodynamic markers, to automatically adjust anesthetic delivery. In a multicenter randomized trial, a bispectral index-guided closed-loop propofol system maintained bispectral index within ±10 of the target for 81.4 ± 8.9% of anesthesia duration compared to 55.3 ± 25% under manual control (*P* < 0.0001).^46^ An observational study has found that these systems also reduce the time patients spend in excessively deep anesthesia (e.g., bispectral index < 40), prevent overventilation, and decrease fluid over-administration.^47^ In regions where end-tidal controlled anesthesia delivery is implemented, such as Europe and Australia, hospitals have observed 44% reductions in greenhouse gas emissions and 27% reductions in operating room costs, with the added benefit of reducing clinician workload by halving manual adjustments.^48^ Although such systems are not yet approved in the United States for automated intravenous drug delivery, they are increasingly integrated into next-generation anesthesia workstations globally. Gas capturing technologies and closed-loop systems reflect two distinct but complementary pathways to improving the environmental sustainability and clinical precision of modern anesthesia practice. Further research is essential to fully quantify their cost-effectiveness, life cycle impact, and long-term feasibility across diverse health system settings. A comparison of gas capturing technologies is presented in **Table 3**.

**Table 3:** Gas capturing technologies

Technology	Mechanism	Reported Capture efficiency	Notes/limitations
CONTRAfluran™ (Baxter)	Adsorption or condensation into liquid	Claims up to 99%, *in vivo* study: 25% for desflurane at 0.8 L/min FGF; 43–51% for isoflurane and sevoflurane	Capture rate strongly influenced by FGF rate; lower capture after patient emergence.
Blue-Zone (Deltasorb®/Centralsorb®)	Zeolite canisters adsorb gases; can be reprocessed into generic anesthetic	*In vitro* > 95%; *in vivo* 25–73% depending on agent & setup	Offers closed-loop recycling; variable efficiency in real-world settings.
Closed-loop anesthesia delivery systems (not a capture tech but complementary)	Automated titration of anesthetics (e.g., BIS- or ET-controlled)	↓ 44% emissions and ↓ 27% OR costs in EU/AU studies	Reduces waste by preventing overdelivery; not yet standard in US.

BIS: Bispectral index; ET: end-tidal; EU/AU: European Union or Australia; FGF: fresh gas flow; OR: operating expenditure.

### Environmental impact of total intravenous anesthesia and regional anesthesia

TIVA presents a common alternative to the practice of general anesthesia with inhalational anesthetics. TIVA does not involve emission of gases and studies have shown that it is more environmentally friendly than inhalational anesthetics.^43^,^49^ Bernat et al.^17^ examined the CO_2_ equivalent footprint of using TIVA versus a mixed strategy of TIVA and inhaled anesthetic. Using TIVA alone lowered the hospital’s carbon footprint 20 times more than the mixed strategy. The researchers hypothesized that using a TIVA-only strategy across Europe could reduce the CO_2_ equivalent emissions similar to those released by a car travelling 7.5 billion km annually.^17^ However, it is very difficult to directly compare the negative environmental effects of TIVA vis-à-vis inhaled agents since TIVA requires intravenous tubing, transport, and waste. Furthermore, albeit the data is limited, propofol has been shown to be quite ecotoxic to aquatic environments due to its low biodegradability, bioaccumulation potential, and toxicity to aquatic life such as neurotoxicity, developmental delay and potential metabolic dysfunction.^50^ Finally, TIVA does not provide the same guarantee of amnesia as the use of potent inhaled anesthetics, increasing the risk for recall (awareness under anesthesia) during surgery.

Regional anesthesia is another alternative that minimizes the amount of medication and equipment needed, along with eliminating the expulsion of anesthetic gases into the atmosphere.^49^ One study in the United Kingdom found that epidural anesthesia lowered carbon emissions 200-fold when compared to N_2_O when either was used in a 4-hour labor.^51^ Researchers from the University of Alberta calculated that using spinal analgesia 96% of the time, rather than inhalational anesthetics, for arthroplasties, could annually save 40–240 tons of CO_2_ equivalent emissions.^52^ Although there are some studies that support regional anesthesia having a lower carbon footprint compared to general anesthesia, wide variation exists in regional anesthetic practices.49 Furthermore, it is extremely difficult to tally the environmental pollution created by regional anesthesia *vs*. regional anesthesia with TIVA *vs*. general anesthesia.

### Economic considerations

One of the main concerns of using TIVA over inhalational anesthetics is the associated costs.^53^ Multiple studies have shown that TIVA is generally more expensive than sevoflurane and isoflurane, but less costly than desflurane. For example, a cost analysis in Colombia reported average per-case costs of approximately USA dollar (USD) 56 for TIVA, compared to USD 32 for sevoflurane, USD 25 for isoflurane, and USD 73 for desflurane, across procedures lasting 1–6 hours.^54^ A similar trend was noted in India, where TIVA had the highest variable cost at roughly ₹2713.82 (approximately USD 30.94), compared to low-flow isoflurane at ₹1981.62 (approximately USD 22.95).^55^ In the United States, propofol infusion for a standard 70-kg patient costs approximately USD 12.75 per hour, compared to sevoflurane, which ranges from USD 3.50 to USD 7.00 per hour depending on FGF and facility-specific pricing.^56^ Collectively, these findings confirm that TIVA consistently incurs higher intraoperative costs relative to sevoflurane and isoflurane, although it may be less expensive than desflurane depending on market and equipment-related variables.

The higher cost of TIVA is largely driven by the price of propofol and other medications, such as remifentanil, the patient’s weight, and the need for infusion pumps and monitoring equipment. In contrast, inhalational agents are administered via vaporizers integrated into standard anesthesia machines, reducing equipment-related expenses.^54^ Despite higher intraoperative costs, TIVA may result in lower postoperative costs due to faster recovery and fewer complications such as post-operative nausea and vomiting, especially if propofol was utilized. One study based in the United States found that TIVA reduced healthcare system costs by 11.41 ± 10.73 USD per patient through decreased post-anesthesia care unit recovery time and medication use, such as antiemetics.^57^ Another study corroborated that TIVA had the lowest post-anesthesia care unit costs due to faster recovery and fewer adverse effects despite the highest intraoperative cost (USD 52.73).^58^

The clinical benefit of TIVA in reducing postoperative nausea and vomiting has been corroborated in multiple trials. For example, a Dutch randomized controlled trial demonstrated that TIVA significantly reduced the incidence of postoperative nausea and vomiting, from 61% to 46% in inpatients and from 46% to 28% in outpatients.^59^ However, the same study also highlighted that intraoperative costs with TIVA were more than three times higher than those associated with an isoflurane-N_2_O regimen. Moreover, although post-anesthesia care unit stays were modestly shorter in the TIVA group, these differences did not translate into measurable recovery cost savings within the hospital accounting framework.^59^ Nonetheless, in the broader economic context—particularly in ambulatory settings where throughput is a critical metric—the cost of managing a single episode of postoperative nausea and vomiting can exceed USD 75, factoring in delays, antiemetics, and unplanned admissions.^60^ This underscores the relevance of evaluating TIVA not merely through drug acquisition costs but in terms of total perioperative economic impact.

### Central N_2_O piping

N_2_O is one of the most environmentally detrimental gases that can be used during anesthesia due to the required large quantities of the gas and its 114-year atmospheric lifetime.^30^ It is common to have central piping infrastructure to deliver N_2_O for clinical use in operating rooms.^31^ However, central piping systems often contain leaks that increase N_2_O release into the environment.61 In the United Kingdom, a 2020 report found that two hospitals had a collective annual 1.5 × 10^6^ L of N_2_O lost due to leaks.^62^ Additional reports that included hospitals in the United Kingdom, New Zealand and the United States found that 77–95% of the N_2_O piped through central systems was lost before being used clinically.^30^ Lastly, a hospital in Melbourne, Australia reported that 83.5% of the N_2_O that was to be used in 35 operations, was lost due to leaks.^62^ If this amount of N_2_O was lost annually, it would equate to 101 tons of CO_2_e.^63^ Not only are these N_2_O leaks environmentally damaging, but they are also associated with increased costs and could cause occupational hazards.^61^ One solution has been to eliminate central N_2_O piping systems in favor of portable N_2_O cylinders, which can significantly decrease the amount of N_2_O lost.^30^,^31^

### Electronic medical record and anesthesia support systems

Low FGF rates are a key factor in reducing excessive use of inhaled anesthetics.^31^ During anesthetic procedures, the selection of FGF rates is user dependent.^64^ Anesthesiologists often worry that low gas flow rates may not provide adequate depth of anesthesia needed for maintenance, leading to larger FGF rates being chosen.^64^ To account for the user dependent aspect of FGF selection, several software programs have been created that can be integrated into intraoperative anesthesia monitoring systems within a hospital’s electronic medical record (EMR).

The Smart Anesthesia Manager™ (SAM, University of Washington, Seattle, USA) is a system that can be embedded into a hospital’s EMR.^65^ SAM adds to the EMR by detecting and notifying practitioners about a variety of issues, including inhaled anesthetic wastage.^66^ SAM detects issues in real-time and provides suggestions for improvement through the intraoperative anesthesia system of the EMR.^66^ The messages are also prioritized by importance so that only three notifications are sent out to minimize alert fatigue. An analysis of SAM showed that annual savings of USD 120,168 were attributed to a reduction in excess FGF.^66^ Another study conducted at a university hospital in Australia using a similar system to automate control of end-tidal concentrations found a decrease in anesthetic gas cost per hour by over 25% as the number of volatile agent bottles used dropped, along with their corresponding acquisition costs.^67^ At the University of California, San Francisco, a comparable clinical decision support system was created in hopes that it could be generalizable to various hospital systems.^66^ The clinical decision support system provides active, real-time intraoperative alerts to practitioners. The alert was designed to be non-interruptive, so that practitioners were not distracted, but with the goal of prompting change. The clinical decision support system was shown to be effective at reducing inhaled anesthetic waste at five University of California Health Network campuses.^66^

The Low Flow Wizard is another system created with the purpose of prompting anesthesiologists to reduce their FGFs.^64^ The Low Flow Wizard determines the most efficient FGF based on the volume of gas delivered versus the volume taken up by the patient. The system will then provide real-time recommendations when FGFs are too high or too low. The Low Flow Wizard was shown to have a median 53.2% reduction in FGF during the maintenance phase of anesthesia for surgical cases that were relatively long.^64^ Lastly, the EMR can be used to hold physicians accountable for the anesthetic drugs they select, the gas volumes used, and FGFs chosen.^31^ Not only is it important to analyze this data and set practice standards, but the information should also be shared amongst care teams. The shared information should include individual provider performance and outcomes of implemented initiatives. In Zuckerberg San Francisco General Hospital, an EMR quality improvement initiative was implemented to encourage providers to reduce the volume used and FGF of sevoflurane.^68^ The hospital found a mean reduction of 58.5% in FGF, which equated to more than a 100% improvement from baseline.^68^ The success of the initiative was partly due to consistent staff presentations regarding the initiative and identifying current top participators.

## Conclusion

Minimizing the impact of our healthcare system on the environment is an important task. Since the operating room is a resource-intensive environment and general anesthesia is becoming increasingly common, it is critical that we understand the environmental impact of anesthetic gases and devise ways to mitigate their negative effects. Many of the waste and impact reduction strategies discussed above are not difficult to implement, but require discussion, education and cultural buy-in at both an institutional and individual level. For instance, many practitioners have grown accustomed to desflurane and its pharmacodynamics and pharmacokinetics over the course of decades in practice. Persuading these practitioners to make a change toward sevoflurane or TIVA would likely require large scale institutional buy-in and education. Similarly, many practitioners have been trained to be wary of low flow anesthesia when using sevoflurane due to the package insert warning about compound A. Recently, the American Society of Anesthesiologists released a statement on the use of low flows with sevoflurane, supporting the use of flows as low as 0.5 L/min with sevoflurane use.^69^

Another challenge in implementing sustainability initiatives is how to measure successful measures. Anesthesia machines may offer the capability to either purchase a software dashboard or manually pull the flow and gas usage data directly from the machines. Some modern anesthesia machines may even go so far as to offer a way to automatically set FGFs and end tidal anesthetic gas concentration at pre-specified levels, thus ensuring gas waste is kept to a minimum. Despite cost savings, purchasing new anesthesia machines with the option of automation may not be feasible at most institutions and may require a relatively long period of time to recoup the initial investment costs. Regardless, additions to EMR modules and anesthesia support systems are noteworthy options that continually prompt and educate staff about anesthetic gas sustainability initiatives. These may be paired with software dashboards or gas usage data, allowing the anesthesiologist to tailor the anesthetic to both patient and surgical procedure, as well as remaining conscientious about the environmental impact of the anesthetic.

Eliminating central N_2_O piping is another reasonable way to decrease gas waste into the environment. Most newly built facilities seem to have moved away from building centrally stored N_2_O tanks and piping. The elimination of central N_2_O presents a logistical challenge in older facilities, a resource intensive measure that requires high level institutional buy-in. Unfortunately, the cost savings in this scenario are slightly less favorable, as N_2_O is typically not a very expensive gas to purchase. Achieving cultural buy-in in this situation is somewhat more difficult, with roots solely in the realm of minimizing environmental impact and occupational exposure.

Gas capture technology presents a promising initiative that would both decrease gas waste into the environment and recycle used agent into pharmacologically active gas ready to be used clinically once again. This technology, though used more extensively in other parts of the world, seems to be in its infancy in the United States. It may be possible to become a trial site for systems such as CONTRAFluran™ (Baxter), but it was not available for purchase and implementation at the time of this research. Overall, while promising and possibly a wave of the future, gas capture technology requires more research and development to become a viable option for waste reduction and recycling.

**Figure 4:**
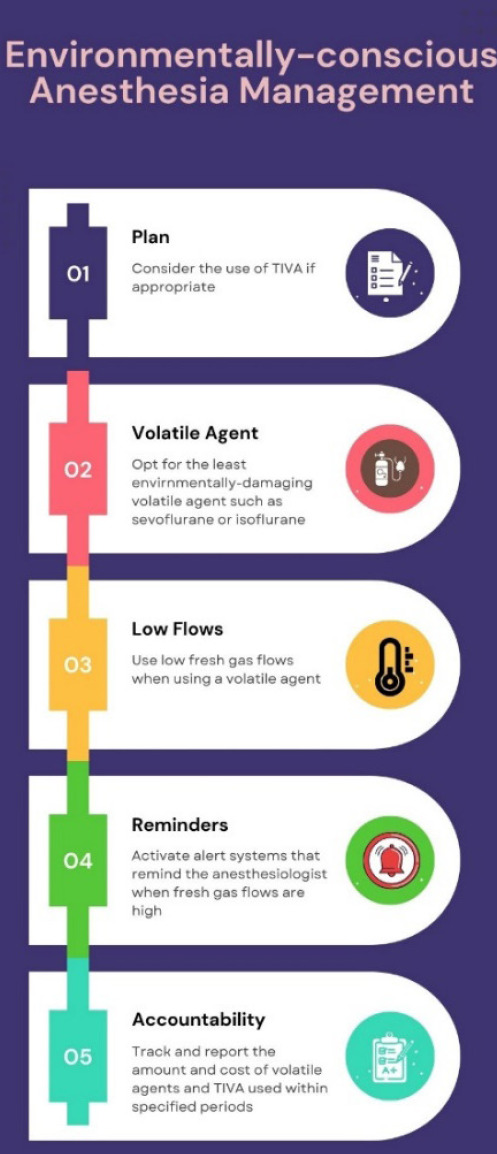
Environmentally-conscious anesthesia management. ©Ana Costa via Canva.com. TIVA: Total intravenous anesthesia.

Limitations of this study include a paucity of robust trials that can truly compare the environmental impact of anesthetic gases with alternative methods of anesthesia, such as TIVA or regional anesthesia. Another limitation is the preponderance of literature on this topic that is applicable to industrialized nations, whereas low-resourced nations are not typically represented in these studies. Additionally, mitigation strategies to minimize the detrimental environmental impact of anesthetic gases on the globe often necessitate additional cost, which low-resourced healthcare facilities may not have, thus limiting their anesthetic agents and equipment to those that represent the lowest cost. However, implementing sustainability initiatives, especially as they pertain to anesthetic gas waste, is an important issue to tackle. Effective change can stem from modifications to practice that are simple to implement, but often difficult to achieve secondary to previously established cultural and institutional norms. Tracking success can sometimes present a challenge, and a clear plan is required to achieve institutional buy-in. A favorable startup cost/return on investment ratio, in addition to the benefit of mitigating environmental impact, is often helpful in convincing individuals and institutions to move toward more sustainable practices. As the world continues to focus more on green initiatives, hopefully these sustainable practices will become more commonplace (**Figure 4**).

## Data Availability

*Not applicable.*
